# A novel predictive model based on preoperative blood neutrophil-to-lymphocyte ratio for survival prognosis in patients with gastric neuroendocrine neoplasms

**DOI:** 10.18632/oncotarget.9805

**Published:** 2016-06-03

**Authors:** Long-Long Cao, Jun Lu, Jian-Xian Lin, Chao-Hui Zheng, Ping Li, Jian-Wei Xie, Jia-Bin Wang, Qi-Yue Chen, Mi Lin, Ru-Hong Tu, Chang-Ming Huang

**Affiliations:** ^1^ Department of Gastric Surgery, Fujian Medical University Union Hospital, Fuzhou, Fujian Province, 350001, People's Republic of China

**Keywords:** gastric neuroendocrine neoplasms, preoperative blood neutrophil-to-lymphocyte ratio, prognosis, tumor recurrence, surveillance strategy

## Abstract

**Purpose:**

Evaluate the predictive value of the preoperative blood neutrophil-to-lymphocyte ratio (NLR) on the clinical outcomes of patients with gastric neuroendocrine neoplasms (g-NENs) after radical surgery.

**Results:**

The NLR was significantly higher in patients with g-NENs than in matched normal volunteers (*P* < 0.05). A higher blood NLR was not significantly associated with clinical characteristics (all *P* > 0.05). According to the multivariate analysis, the NLR was an independent prognostic factor of RFS and OS. Nomograms, including the NLR, Ki-67 index and lymph node ratio, had superior discriminative abilities to predict clinical outcomes. The recurrence rate was 37% (55/147). The median time to recurrence was 9 months; 48 (87%) patients experienced recurrence within the first 2 years. Both the NLR and Ki-67 index were correlated with liver metastases (both *P* < 0.05) and were also negatively correlated with recurrence time (both *P* < 0.05).

**Materials And Methods:**

We enrolled 147 patients who were diagnosed with g-NENs and underwent radical surgery. Receiver operating characteristic curve analysis was used to identify the optimal value for blood NLR. Univariate and multivariate survival analysis were used to identify prognostic factors for g-NENs. A nomogram was adopted to predict RFS and OS after surgery.

**Conclusions:**

As an independent prognostic factor for g-NENs, blood NLR can improve the predictability of RFS and OS. We recommend that g-NEN patients with a high blood NLR or high Ki-67 index undergo surveillance during the first month and then every 3 months for 2 years post-surgery.

## INTRODUCTION

Gastric neuroendocrine neoplasms (g-NENs) are a type of relatively rare tumors mainly derived from enterochromaffin-like cells (ECL-cells) localized in the gastric mucosa [1]. Although increasingly recognized due to the expanding indications for upper gastrointestinal (UGI) endoscopy, g-NENs are still poorly understood tumors with inconsistent clinicopathological and biological characteristics [2, 3]. Apart from early diagnosis, the most important component of proper management is identifying the prognostic factors for g-NEN patients. Based on the National Comprehensive Cancer Network (NCCN) guidelines [4, 5], the TNM scoring system includes the depth of invasion and lymph node metastases as very important prognostic factors for patients with g-NENs. The guidelines recommend that patients with g-NENs should be reevaluated 3 to 12 months after resection and then every 6 to 12 months for up to 10 years. However, the prognostic factors and surveillance strategy for patients with g-NENs have not been clearly defined due to the complexity and rarity of this disease [6, 7]. In recent years, the preoperative blood neutrophil-to-lymphocyte ratio (NLR) is considered to be significantly associated with oncological outcomes [8, 9], and an elevated NLR has been correlated with advanced stage and poor prognosis in a variety of human tumors, including colorectal cancer [10], gastric adenocarcinoma [11], non–small cell lung cancer [8], and hepatocellular carcinoma [12]. However, little research has focused on the relationship between blood NLR and the prognosis of patients with neuroendocrine neoplasms, particularly g-NENs. We evaluated blood NLR as a prognostic indicator and investigated its clinical value for postoperative surveillance in patients undergoing radical surgery for g-NENs.

## RESULTS

### Blood NLR was elevated in patients with g-NENs

**Figure 1:**
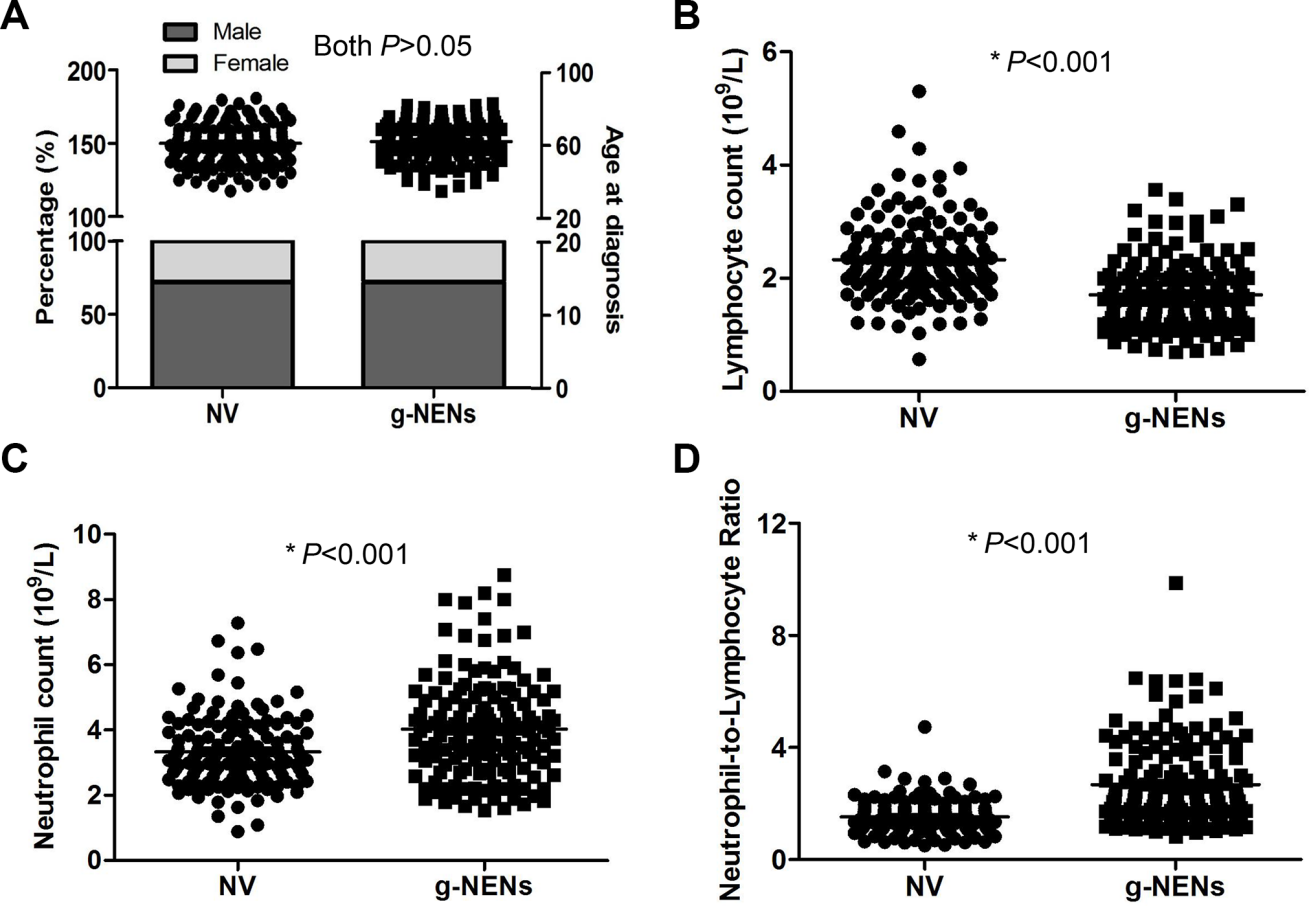
Blood cell counts from normal volunteers and gastric neuroendocrine neoplasms patients (**A**) A total of 147 NVs were one-to-one matched by age and gender. There were no differences in age or gender between the NV and the g-NEN groups (both *P* > 0.05). (**B**) The lymphocyte counts were significantly lower in the g-NEN group than in the NV group (1.71 ± 0.05 vs 2.33 ± 0.06, *P* < 0.001). (**C**) The neutrophil counts from g-NEN patients were significantly higher than those of the NVs (4.03 ± 0.13 vs 3.33 ± 0.09, *P* < 0.001). (**D**) Blood NLR in the g-NEN group was significantly higher than in the NV group (2.67 ± 0.13 vs 1.52 ± 0.05, *P* < 0.001).

In total, 147 normal volunteers (NVs) with similar age and gender proportions as the neoplasm patients were enrolled (both *P* > 0.05, Figure 1A). The lymphocyte counts were significantly lower in the blood of patients with g-NENs than in NVs (*P* < 0.001, Figure 1B). The neutrophil counts and NLR were significantly higher in the patients than in the NVs (both *P* < 0.001, Figure 1C and 1D).

### An elevated blood NLR was not associated with unfavorable clinicopathologic factors

The univariate analysis revealed (Table 1) that a high blood NLR was associated with large tumor size, high Ki-67 index, invasion depth, high lymph node ratio (LNR), and histological type (all *P* < 0.05). However, the multivariate analysis revealed no significant differences in the above clinicopathological factors between the two groups (all *P* > 0.05).

**Table 1:** Characteristics of 147 patients with g-NENs between different blood neutrophil-to-lymphocyte ratios

Clinicopathological features	Blood NLR		Univariable analysis	Multivariable analysis
	≤ 2.20 (*n* = 77)	> 2.20 (*n* = 70)	*P* values	*P* values
Age (Y)			0.193	
≤ 70	63	51		
> 70	14	19		
Gender			0.575	
Male	54	52		
Female	23	18		
Tumor site			0.052	
Upper	40	28		
Middle	12	14		
Lower	21	15		
Mixed	4	13		
Tumor size (cm)			< 0.001^*^	0.080
≤ 3.5	38	12		
> 3.5	39	58		
Ki-67 index (%)			0.001^*^	0.675
≤ 2	17	2		
≥ 3, ≤ 20	14	10		
> 20	46	58		
Depth of invasion			< 0.001^*^	0.192
T1	22	1		
T2	6	5		
T3	31	34		
T4	18	30		
Lymph node ratio			0.001^*^	0.079
0	28	11		
> 0, ≤ 0.2	31	21		
> 0.2, ≤ 0.4	9	25		
> 0.4	9	13		
Lymphovascular invasion			0.513	
No	47	39		
Yes	30	31		
Histological type			0.035^*^	0.621
NET	20	7		
NEC	21	27		
MANEC	36	36		
ASA status			0.337	
1 + 2	69	59		
3 + 4	8	11		
Postoperative complication			0.587	
No	57	49		
Yes	20	21		

### Elevated blood NLR was associated with poor prognosis

**Figure 2:**
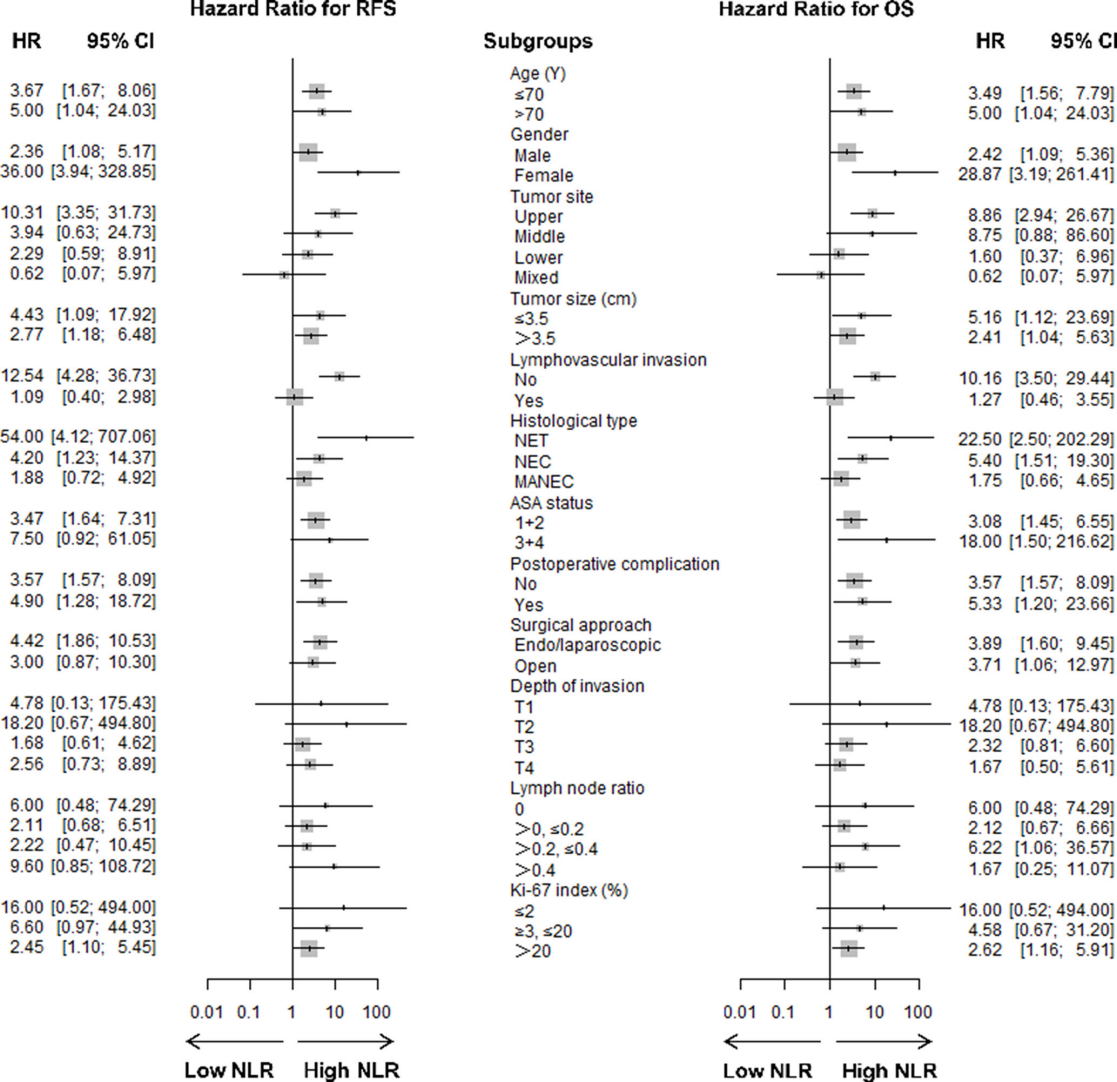
Forest plot showing hazard ratios (oblongs) and 95% CI (bars) for RFS (left) and OS (right) (according to subgroups) among 147 g-NENs patients undergoing radical surgery Long-term survival, including RFS and OS, was better among patients with low blood NLR than among patients with high blood NLR. HR = hazard ratio, CI = confidence interval.

As shown in Figure 2, the RFS and OS were analyzed according to age, gender, tumor site and size, lymphovascular invasion, histological type, ASA status, postoperative complications, surgical approach, invasion depth, LNR, and Ki-67 index. The hazard ratio and 95% confidence interval for RFS and OS were compared among the subgroups. The long-term survivals, including RFS and OS, were poorer in the high blood NLR group than in the low blood NLR group.

### Blood NLR, combined with the Ki-67 index and LNR, was a superior prognosis predicting system

To investigate which parameters were associated with clinical outcomes, a univariate survival analysis and a multivariate survival analysis for RFS and OS were performed. The univariate analysis identified larger tumor size, presence of postoperative complications, greater invasion depth, higher LNR, higher Ki-67 index, and higher blood NLR as prognostic indicators for poorer RFS (all *P* < 0.05, Table 2). The tumor size, invasion depth, LNR, Ki-67 index, and blood NLR were identified as prognostic indicators for OS (all *P* < 0.05, Table 3). According to the multivariate analysis, the Ki-67 index, LNR, and blood NLR were independent prognostic factors for RFS and OS (all *P* < 0.05, Table 2 and Table 3).

**Table 2:** Variables associated with recurrence-free survival according to the Cox proportional hazards regression model

Variables	Univariable analysis			Multivariable analysis		
	Hazard ratio	95% CI	*P*	Hazard ratio	95% CI	*P*
Age (Y)			0.865			
≤ 70	Reference					
> 70	1.052	0.587 to 1.884				
Gender			0.135			
Male	Reference					
Female	0.627	0.339 to 1.157				
Tumor site			0.744			
Upper	Reference					
Middle	0.751	0.356 to 1.585				
Lower	0.908	0.488 to 1.688				
Mixed	1.289	0.566 to 2.938				
Tumor size (cm)			0.008^*^			0.245
≤ 3.5	Reference			Reference		
> 3.5	2.362	1.255 to 4.444		NA	NA	
Clinical classification			0.115			
Type 1	Reference					
Type 2	6.397	0.747 to 54.814				
Type 3	9.429	1.206 to 73.708				
Type 4	10.054	1.385 to 72.968				
Histological type			0.121			
NET	Reference					
NEC	2.257	1.013 to 5.029				
MANEC	1.611	0.736 to 3.526				
ASA status			0.222			
1 + 2	Reference					
3 + 4	1.503	0.782 to 2.889				
Postoperative complication			0.041^*^			0.215
No	Reference			Reference		
Yes	1.768	1.023 to 3.053		NA	NA	
Surgical approach			0.209			
Endo/laparoscopic	Reference					
Open	0.719	0.429 to 1.203				
Depth of invasion			0.006^*^			0.546
T1	Reference			Reference		
T2	8.125	0.845 to 78.172		NA	NA	
T3	12.443	1.687 to 91.781		NA	NA	
T4	20.671	2.819 to 151.578		NA	NA	
Lymph node ratio			< 0.001^*^			< 0.001^*^
0	Reference			Reference		
> 0, ≤ 0.2	6.003	1.789 to 20.141		4.213	1.237 to 14.347	
> 0.2, ≤ 0.4	8.714	2.588 to 29.337		4.557	1.320 to 15.734	
> 0.4	18.324	5.350 to 62.765		15.506	4.474 to 53.746	
Ki-67 index (%)			0.003*			0.014^*^
≤ 2	Reference			Reference		
≥ 3, ≤ 20	3.024	0.641 to 14.254		1.827	0.369 to 9.054	
> 20	7.082	1.721 to 29.145		3.993	0.923 to 17.281	
Blood NLR			< 0.001*			< 0.001^*^
≤ 2.20	Reference			Reference		
> 2.20	3.457	2.020 to 5.917		2.751	1.572 to 4.813	

**Table 3:** Variables associated with overall survival according to the Cox proportional hazards regression model

Variables	Univariable analysis			Multivariable analysis		
	Hazard ratio	95% CI	*P*	Hazard ratio	95% CI	*P*
Age (Y)			0.496			
≤ 70	Reference					
> 70	1.228	0.679 to 2.221				
Gender			0.175			
Male	Reference					
Female	0.643	0.339 to 1.218				
Tumor site			0.178			
Upper	Reference					
Middle	0.604	0.277 to 1.315				
Lower	0.557	0.273 to 1.138				
Mixed	1.337	0.588 to 3.043				
Tumor size (cm)			0.003*			0.807
≤ 3.5	Reference			Reference		
> 3.5	2.943	1.441 to 6.009		NA	NA	
Clinical classification			0.116			
Type 1	Reference					
Type 2	4.576	0.510 to 41.017				
Type 3	8.281	1.048 to 65.435				
Type 4	8.809	1.211 to 64.084				
Histological type			0.141			
NET	Reference					
NEC	2.291	0.982 to 5.345				
MANEC	1.652	0.716 to 3.808				
ASA status			0.160			
1 + 2	Reference					
3 + 4	1.633	0.823 to 3.241				
Postoperative complication			0.382			
No	Reference					
Yes	1.313	0.713 to 2.418				
Surgical approach			0.227			
Endo/laparoscopic	Reference					
Open	0.719	0.421 to 1.228				
Depth of invasion			0.020*			0.523
T1	Reference			Reference		
T2	8.653	0.899 to 83.244		NA	NA	
T3	11.637	1.572 to 86.134		NA	NA	
T4	17.657	2.397 to 130.061		NA	NA	
Lymph node ratio			<0.001*			<0.001*
0	Reference			Reference		
> 0, ≤ 0.2	5.302	1.567 to 17.937		3.753	1.087 to 12.961	
> 0.2, ≤ 0.4	7.282	2.144 to 24.731		4.115	1.175 to 14.409	
> 0.4	16.085	4.655 to 55.584		11.412	3.208 to 40.594	
Ki-67 index (%)			0.003^*^			0.011^*^
≤ 2	Reference			Reference		
≥ 3, ≤ 20	2.523	0.524 to 12.155		1.348	0.257 to 7.078	
> 20	6.666	1.613 to 27.553		3.628	0.820 to 16.047	
Blood NLR			< 0.001^*^			0.005^*^
≤ 2.20	Reference			Reference		
> 2.20	3.418	1.925 to 6.068		2.334	1.286 to 4.237	

**Figure 3:**
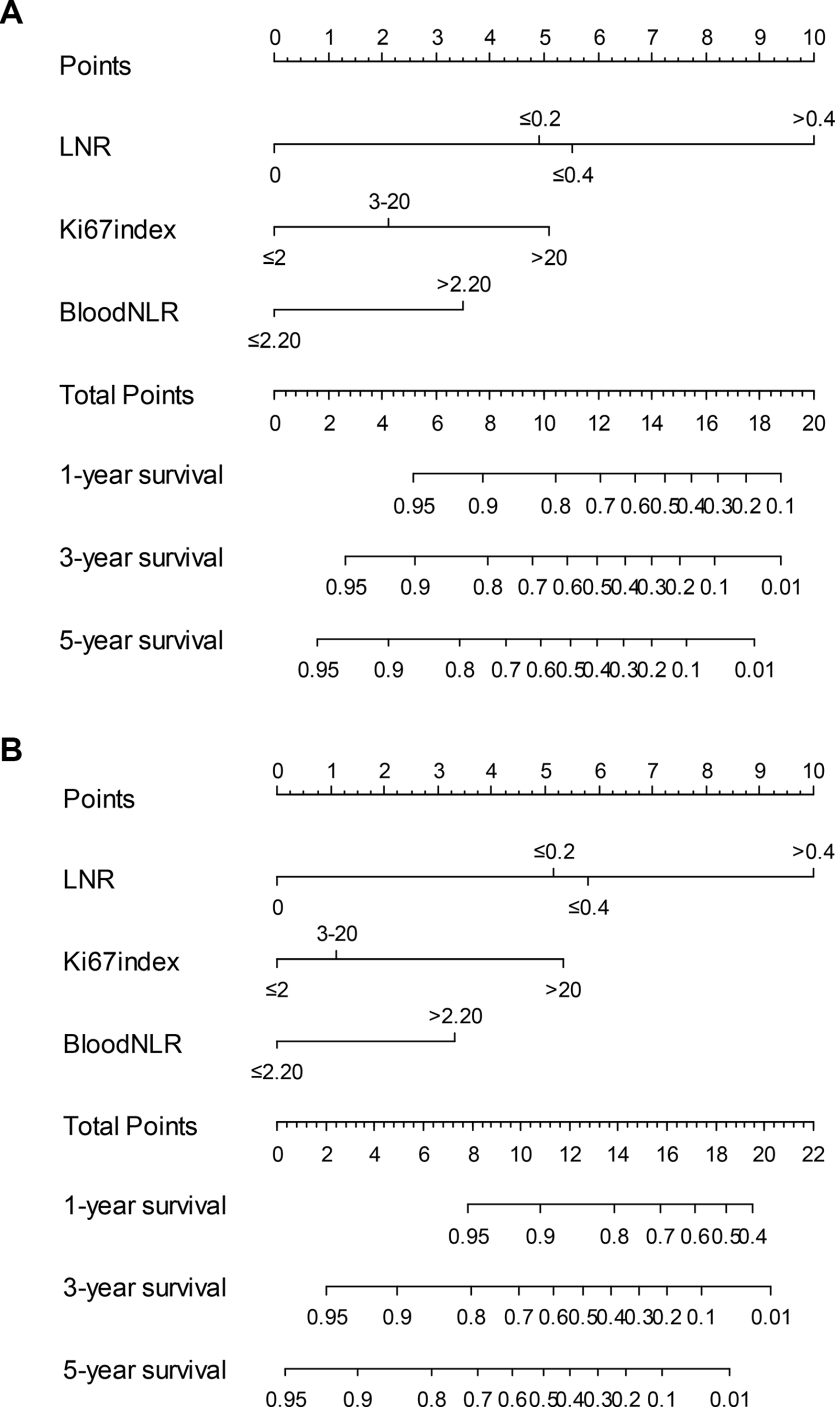
Nomograms for predicting recurrence free survival (A) and overall survival (B) of patients following g-NEN resection LNR = lymph node ratio.

Prognostic nomograms and its calibration curve were established with the R software (Figure 3 and Supplementary Figure, respectively). The C-index of the nomograms for RFS (OS) with blood NLR, LNR, or Ki-67 index were 0.663 (0.652), 0.709 (0.695), and 0.630 (0.628), respectively. However, the C-index of nomograms for RFS (OS), including all three variables, were up to 0.776 (0.760). We also calculated the C-index of the TNM staging system for RFS (OS), which was 0.678 (0.667). All these data indicated that the nomograms, including blood NLR, LNR, or Ki-67 index, had a superior ability to predict clinical outcomes for patients with g-NENs, as well as the traditional TNM staging system.

### Blood NLR and the Ki-67 index were both significantly correlated with recurrence site and recurrence time

**Figure 4:**
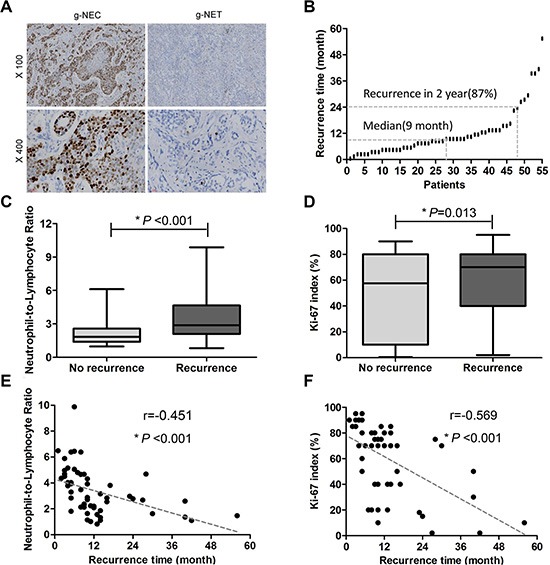
The relationships among Ki-67 index, blood NLR and tumor recurrence (**A**) Representative immunohistochemical staining for Ki-67. (**B**) Fifty-five patients experienced tumor recurrence. The median time to recurrence was 9 (range 1–56) months, and 87% (48/55) patients recurred within the first 2 years. (**C**) Significant differences in blood NLR were observed between the recurrence group and the non-recurrence group (3.36 ± 0.24% vs 2.26 ± 0.12%, mean ± SEM. *P* < 0.001). (**D**) The Ki-67 index was significantly higher in the recurrence group (60.67 ± 3.66%) versus the non-recurrence group (47.23 ± 3.49%, *P* = 0.013). (**E**) The blood NLR was inversely correlated with time to recurrence (*r* = −0.451, *P* < 0.001). (**F**) The Ki-67 index inversely correlated with time to recurrence (*r* = −0.569, *P* < 0.001).

The recurrence rate was 37% (55/147). The median time to recurrence was 9 (range 1–56) months, and 87% (48/55) of patients experienced recurrence within the first 2 years, and only 7% (4/55) of patients experienced recurrence > 3 years after surgery (Figure 4B). As shown in Figure 4C and 4D, both the blood NLR and Ki-67 index were significantly higher in the recurrence group than in the non-recurrence group (both *P* < 0.05). Both the blood NLR and Ki-67 index were negatively correlated with the recurrence time (both *P* < 0.05, Figure 4E and 4F).

Details regarding the recurrence site following surgery were listed in Table 4. The recurrence rate in the high blood NLR group was significantly higher than in the low blood NLR group (*P* < 0.001). Similar results for recurrence rate were also observed between the high Ki-67 index group and the low and intermediate Ki-67 index groups (*P* = 0.023). Additionally, elevated blood NLR was significantly associated with both liver metastasis and peritoneal metastasis (both *P* < 0.05), whereas only liver metastasis was significantly correlated with a high Ki-67 index (*P* = 0.008).

**Table 4:** Site of recurrence after surgery

Site of recurrence	Blood NLR			Ki-67 index (%)		*P*
	≤ 2.20 (*n* = 77)	> 2.20 (*n* = 70)	*P*	≤ 20 (*n* = 43)	> 20 (*n* = 104)	
Liver	10	29	< 0.001^*^	5	34	0.008^*^
Peritoneal cavity	3	12	0.008^*^	2	13	0.232
Lynph node	4	9	0.102	2	11	0.347
Lung	3	4	0.709	0	7	0.106
Bone	2	4	0.425	1	5	0.671
Adrenal gland	2	3	0.669	0	5	0.322
Pancreas	1	3	0.347	1	3	1.000
Locoregional recurrence	2	3	0.669	2	3	0.630
Spleen	0	2	0.225	1	1	0.501
Kidney	0	2	0.225	0	2	1.000
Brain	1	0	1.000	0	1	1.000
Number of patients with recurrence	16	39	< 0.001^*^	10	45	0.023^*^

## DISCUSSION

Neuroendocrine neoplasms, particularly g-NENs in the digestive system, are a unique subgroup of tumors with great clinical heterogeneity and varied biology. Recently, the incidence of g-NENs, which account for 6% of neuroendocrine neoplasms in all systems, has gradually increased due to improvements in diagnostic techniques and increasing knowledge regarding g-NENs [13]. In a literature review, the 5-year survival rate of g-NENs was 30–43.8% [14–16]. Data regarding the clinical characteristics and biological tumor characteristics were significantly associated with g-NEN patients' prognosis [17–19]. However, the independent prognostic factors for g-NEN patients are still controversial. To our knowledge, there are still no reports regarding individual prognosis prediction models for g-NENs. This study explored the independent prognostic factors of g-NENs to facilitate the development of a tumor prognosis prediction model to provide individualized therapy and follow-up.

In recent years, pathological staging and the Ki-67 index were viewed as valuable prognostic factors for patients with g-NENs. Deep tumor invasion, the presence of lymph node metastasis and distant metastasis, and a high Ki-67 index were associated with decreased long-term survival [6, 7, 20]. In this study, the rate of lymph node metastasis and the Ki-67 index were independent risk factors for OS and RFS in patients with g-NENs, which was consistent with previous reports. Although there were obvious prognostic difference between NET patients and NEC patients, histological type was not a prognostic factor in the study. Small sample sizes and short-term following up in the study may explain the problem. In addition, increasing evidence has confirmed that the systemic inflammatory response is closely related to the prognosis of malignant tumors [21–23], but the relationship between the systemic inflammatory response and g-NENs has been unclear. Our study was the first to compare blood NLR between patients with g-NENs and NVs. The blood NLR in the g-NENs patients was higher than in the NVs. We then observed, through forest Figures, that a high blood NLR was significantly associated with a poor prognosis, and a multivariate analysis further revealed that blood NLR was an independent risk factor for patients with g-NENs. Currently, nomograms, a new type of statistical prediction model, have been developed for the majority of cancer types [24, 25]. Prognostic nomograms are useful and are relatively easy to read with simple graphics. Nomograms enable a combination of multiple relevant clinical predictors and can be applied to individual patients. For many cancers, nomograms compare favorably to the traditional TNM staging systems and thus have been proposed as an important tool in clinical practice [26, 27]. In this study, we established prognostic nomograms for g-NENs by combining blood NLR, the Ki-67 index, and LNR, and the C-index in the combination was higher than using any one parameter alone. Furthermore, this combination had a high predictive ability as well as the traditional TNM staging system. Therefore, the combination of blood NLR, the Ki-67 index and LNR, as a novel prognostic system, may provide simple, more accurate prognosis predictions.

Postoperative local recurrence and distant metastasis are the leading causes of death for malignant tumor patients. In this study, the 5 years recurrence rate for patients with g-NENs after radical surgery was 37%. Liver metastasis was the most common type of recurrence, whereas the spleen, kidney, and brain were relatively rare sites of recurrence. Our results were similar to previous reports [7]. In the present study, both the Ki-67 index and blood NLR were closely related to the site of tumor recurrence and a high incidence of liver metastasis was observed in patients with a high blood NLR or high Ki-67 index. In addition, in patients who had recurrence, both the blood NLR and Ki-67 index had strong inverse relationships with the time to recurrence. Thus, during the process of postoperative follow-up, clinicians should recognize the diagnostic and prognostic value of the Ki-67 index. To discover potential hepatic metastases earlier, clinicians should also recognize the predictive value of tumor recurrence based on the preoperative blood NLR.

As most of the current guidelines recommend, postoperative surveillance for g-NENs begin at 3 to 12 months after radical surgery and the intervals vary from 6 to 12 months [4, 5, 28]. In the present group, 37 (67%) and 48 (87%) patients with recurrence suffered a tumor relapse within the first and second year after primary resection, respectively. The earliest time of tumor recurrence was only one month after surgery. One patient experienced recurrence at 5 years after surgery. According to the guidelines for postoperative surveillance, starting at 3 months with intervals of 6 months after surgery, 24 (44%) patients in this study would have had delayed tumor identification by at least four months, which implies that these surveillance strategies are not the most effective measures to identify early tumor recurrence. Based on these findings, we recommend that patients with g-NENs and a high blood NLR or Ki-67 index should be closely monitored at 3 month intervals starting at the first month post-surgery for the first 2 years after radical surgery to identify tumor recurrence more quickly.

This study had some limitations. This study was uncontrolled and performed within a single institution. The results should be confirmed by subsequent prospective studies. There were some heterogeneity in this study, such as histological type (including NET, NEC and MANEC). Through accumulating more cases, we will focus on just one of these three types in the future. However, to our knowledge, our study enrolled more patients with g-NENs than similar literature reports and demonstrated that the blood NLR was able to predict long-term survival relatively accurately for patients for the first time. Our study could be the basis for a subsequent prospective clinical study.

In conclusion, as a simple, easily measurable, and inexpensive inflammatory biomarker, a preoperative blood NLR is an independent predictor of RFS and OS, and its combination with the Ki-67 index and LNR could improve prognosis prediction in g-NENs patients undergoing radical surgery. For patients with a blood NLR > 2.20 or a Ki-67 index > 20%, we recommend a modified surveillance strategy starting at the first month post-surgery and at intervals of 3 months for the first 2 years after surgical resection.

## MATERIALS AND METHODS

### General conditions

**Figure 5:**
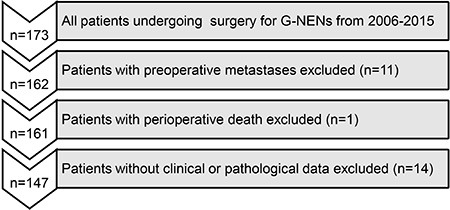
Flow diagram of patient inclusion and exclusion

A total of 173 patients diagnosed with g-NENs at Fujian Medical University Union Hospital between March 2006 and March 2015 were identified from a prospective database. The exclusion criteria for this study were as follows (Figure 5): metastatic disease confirmed preoperatively or at surgery (*n* = 11), perioperative death (n = 1), and incomplete/inaccurate medical records (*n* = 14). A total of 147 patients who underwent curative-intent surgery for g-NENs were enrolled in this study. The pathological data for these patients were reconfirmed by two pathologists according to the North American Neuroendocrine Tumor Society (NANETS) guidelines (2010) [28]. In total, 27 (18.4%) patients were diagnosed with gastric neuroendocrine tumors (g-NETs), 48 (32.7%) with gastric neuroendocrine carcinoma (g-NEC), and 72 (48.9%) with gastric mixed adenoneuroendocrine carcinoma (g-MANEC). Among these patients, 97 (66.0%) received adjuvant chemotherapy. A total of 147 NVs were also enrolled in the study. The ethics committee of Fujian Union Hospital approved this retrospective study. Written consent was obtained from the patients, and their information was stored in the hospital database and used for research.

The preoperative blood cell counts that were collected closest to the date of surgery were obtained from the electronic patient record system in our hospital. The blood NLR was calculated as the absolute neutrophil count divided by the absolute lymphocyte count. A Receiver operating characteristic (ROC) curve analysis was performed in relation to the presence of recurrence and death from any cause. For all 147 patients, a blood NLR of 2.20 had the highest sensitivity and specificity for both outcomes. Therefore, patients were categorized into two groups: low blood NLR (≤ 2.20) with 77 patients and high blood NLR (> 2.20) with 70 patients.

### Immunohistochemistry analysis

Immunohistochemical staining for Ki-67 was performed using formalin-fixed, paraffin-embedded tumor tissue sections (3 μm thick) from 147 g-NENs (Figure 4A). Briefly, the slides were baked at 65°C for 2 hours, deparaffinized with xylene, and rehydrated in graded alcohol. The slides were subjected to antigen retrieval via the high pressure method using antigen retrieval solution. Endogenous peroxidase was inactivated with 3% H_2_O_2_ in methanol. The nonspecific binding was blocked by incubation using 1% bovine serum albumin (BSA; Sigma-Aldrich; St. Louis, MO) in phosphate buffered saline (PBS). Subsequently, the slides were incubated overnight at 4°C with primary monoclonal mouse antibody against Ki-67 (1:100 dilution; Dako; Carpinteria, CA, USA). Normal goat serum was used as a negative control. After washing with PBS, tissue sections were incubated with secondary antibody for 20 minutes at room temperature and then stained with diaminobenzidine (DAB). Finally, the slides were counterstained with hematoxylin, dehydrated, and mounted with a coverslip. Two pathologists blinded to the clinical data reviewed the immunoreactivity for Ki-67 protein under a light microscope. The Ki-67 index was scored according to the percentage of stained tumor cells based on 2000-cell counts [29] and was classified as low (≤ 2% positive cells), intermediate (3% to 20% positive cells), or high (> 20% positive cells). Overall, 19 (12.9%), 24 (16.3%) and 104 (70.8%) patients had a low (≤ 2%), intermediate (3~20%) and high (> 20%) Ki-67 index, respectively.

### Postoperative follow-up

The patients were monitored after surgery by telephone calls, outpatient visits and letters. The overall survival (OS) time was calculated as the number of months from the date of surgery to the date of last contact, date of death for any cause, or date of end point. The recurrence free survival (RFS) time was calculated as the number of months from the date of surgery to the date of identification of disease recurrence (either radiological or histological), the date of death or last contact, or the date of end point. Our department follows a standardized surveillance protocol and follows up with patients at three-month intervals for the first two years, six-month intervals for years two to five, and at least once per year five years after surgery. The postoperative follow-up data included clinical symptoms and signs, laboratory tests, imagological examinations, and endoscopy and biopsy results. In this study, the median follow-up was 40 (range 2–106) months. The median survival time was 25 (range 2–106) months. The median RFS time was 21 (range 1–106) months.

### Construction and validation of the nomogram

On the basis of the results of the multivariable analysis, a nomogram was formulated by R 3.2.0 (http://www.r-project.org) with the survival and rms package. A final model was selected using a backward step-down process, which used the Akaike information criterion as a stopping rule. The model performance for predicting outcome was evaluated by calculating the concordance index (C-index). Calibration of the nomogram for 1-, 3-, and 5-year RFS and OS was performed by comparing the predicted survival with the observed survival after bias correction. Bootstraps with 1,000 resample were used for these activities.

### Statistical analysis

All enumeration and measurement data were analyzed using SPSS 17.0 for Windows (SPSS, Chicago, IL). Chi-square, Fisher's exact or unpaired Student's t tests were used to compare the difference between blood NLR and the clinicopathologic factors, as appropriate. A Pearson correlation was utilized to evaluate the relationship among blood NLR, Ki-67 index and recurrence. Univariate survival analysis was performed using the Kaplan-Meier method, and a log-rank test was used to assess the significance between groups. A multivariate survival analysis was performed using the Cox proportional hazards model. The significant variables from the univariate analysis were included in the model. *P* < 0.05 was considered to be significant.

## SUPPLEMENTARY FIGURE


