# Supplementary material for: A novel predictive model based on preoperative blood neutrophil-to-lymphocyte ratio for survival prognosis in patients with gastric neuroendocrine neoplasms
Source: Oncotarget. 2016 Jun 3;7(27):42045–58. doi: 10.18632/oncotarget.9805 (PMC5173115; doi:10.18632/oncotarget.9805)
Supplement: Supplementary file 1 [file oncotarget-07-42045-s001.pdf]

A novel predictive model based on preoperative blood neutrophil-to-lymphocyte ratio for survival prognosis in patients with gastric neuroendocrine neoplasms

Supplementary Materials

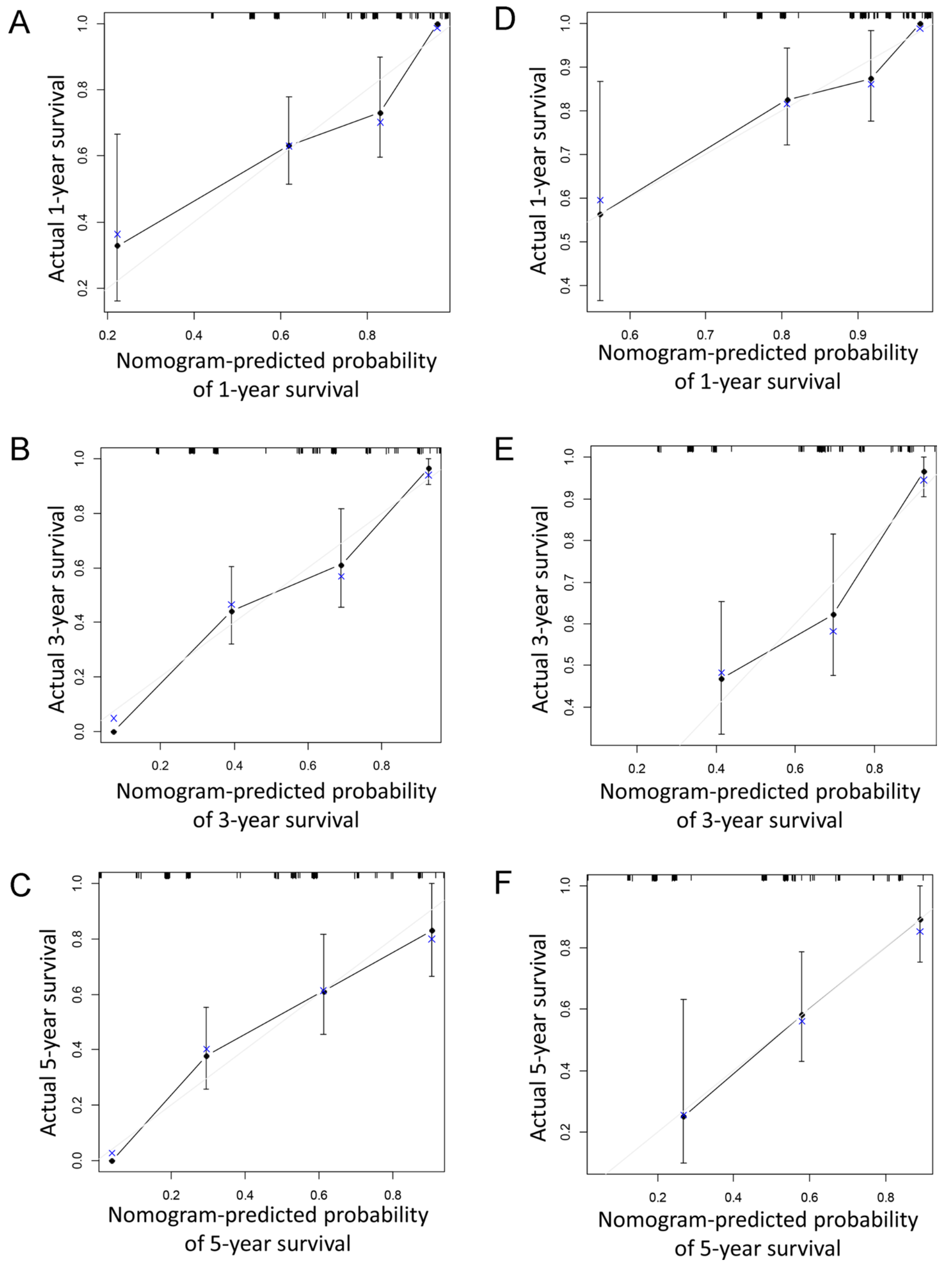

Supplementary Figure S1: The calibration curve for predicting patients recurrence-free survival and overall survival at 1 year (A and D), 3 years (B and E) and 5 years (C and F). Nomogram-predicted probability of overall survival is plotted on the x-axis; actual overall survival is plotted on the y-axis.
